# Differential Synaptic and Extrasynaptic Glutamate-Receptor Alterations in Striatal Medium-Sized Spiny Neurons of Aged YAC128 Huntington’s Disease Mice

**DOI:** 10.1371/currents.hd.34957c4f8bd7cb1f5ec47381dfc811c3

**Published:** 2014-05-21

**Authors:** Eliã P. Botelho, Elizabeth Wang, Jane Y. Chen, Sandra Holley, Veronique Andre, Carlos Cepeda, Michael S. Levine

**Affiliations:** Intellectual and Developmental Disabilities Research Center, Semel Institute for Neuroscience and Human Behavior, Brain Research Institute, The David Geffen School of Medicine, University of California Los Angeles, Los Angeles, California, USA; Intellectual and Developmental Disabilities Research Center, Semel Institute for Neuroscience and Human Behavior, Brain Research Institute, The David Geffen School of Medicine, University of California Los Angeles, Los Angeles, California, USA; Intellectual and Developmental Disabilities Research Center, Semel Institute for Neuroscience and Human Behavior, Brain Research Institute, The David Geffen School of Medicine, University of California Los Angeles, Los Angeles, California, USA; Intellectual and Developmental Disabilities Research Center, Semel Institute for Neuroscience and Human Behavior, Brain Research Institute, The David Geffen School of Medicine, University of California Los Angeles, Los Angeles, California, USA; Intellectual and Developmental Disabilities Research Center, Semel Institute for Neuroscience and Human Behavior, Brain Research Institute, The David Geffen School of Medicine, University of California Los Angeles, Los Angeles, California, USA; Intellectual and Developmental Disabilities Research Center, David Geffen School of Medicine, University of California, Los Angeles, California, USA; Intellectual and Developmental Disabilities Research Center, David Geffen School of Medicine, University of California Los Angeles, California, USA

## Abstract

Huntington’s disease (HD) is a late-onset, slowly progressing neurodegenerative disorder caused by an expansion of glutamine repeats. The YAC128 mouse model has been widely used to study the progression of HD symptoms, but little is known about synaptic alterations in very old animals. The present experiments examined synaptic properties of striatal medium-sized spiny neurons (MSNs) in 16 month-old YAC128 mice. These mice were crossed with mice expressing enhanced green fluorescent protein (EGFP) under the control of either D1 or D2 dopamine receptor promoters to identify MSNs originating the direct and indirect pathways, respectively. The input-output curves of evoked excitatory postsynaptic currents mediated by activation of the α-amino-3-hydroxy-5-methyl-4-isoxazolepropionic acid (AMPA) or N-methyl-D-aspartate (NMDA) receptors were reduced in MSNs in both pathways. In the presence of DL-threo-β-Benzyloxyaspartic acid (DL-TBOA), a glutamate transporter blocker used to increase activation of extrasynaptic receptors, NMDA receptor-mediated currents displayed altered amplitudes, longer decay times, and greater charge (response areas) in both direct and indirect pathway MSNs in YAC128 mice compared to wildtype controls. Amplitudes were significantly increased, primarily in direct pathway MSNs while normalized areas were significantly increased only in indirect pathway MSNs, suggesting that the two types of MSNs are affected in different ways. It may be that indirect pathway neurons are more susceptible to changes in glutamate transport. Taken together, the present findings demonstrate differential alterations in synaptic versus extrasynaptic NMDA receptors in both direct and indirect pathway MSNs in late HD, which may contribute to the dysfunction and degeneration in both pathways.

## Introduction

Huntington’s disease (HD), a slowly progressing neurodegenerative disorder caused by an expansion of glutamine (CAG) repeats, is characterized by motor, psychiatric and cognitive symptoms^1^. The YAC128 mouse model has been widely used to examine mechanisms of HD. YAC128 mice carry the entire human HD gene containing 128 CAG repeats on a yeast artificial chromosome (line 53) and express full-length human mutant huntingtin protein^2^. These mice display a hyperkinetic phenotype at 3 months of age, followed by motor deficits at 6 months, with eventual progression to hypokinesia by 12 months^3^. The behavioral changes are associated with biphasic alterations in excitatory synaptic transmission to striatal medium-sized spiny neurons (MSNs) of the direct (expressing dopamine D1 receptors) and indirect (expressing dopamine D2 receptors) striatal output pathways^4^^,^ 5. At the early, pre-symptomatic stage, glutamate transmission is increased in both direct and indirect pathways. In contrast, at 12 months of age, the more symptomatic phase, glutamatergic transmission is markedly reduced in direct pathway MSNs, while only a trend towards a reduction in glutamatergic inputs is observed in D2 MSNs^4^. Such biphasic alterations are also observed in evoked α-amino-3-hydroxy-5-methyl-4-isoxazolepropionic acid receptor (AMPAR)-mediated currents, demonstrated by an increase in the peak amplitude in indirect pathway MSNs at 1.5 months and a reduction in direct pathway MSNs at 12 months^4^. Alterations in synaptic communication lead to a progressive disconnection between cortex and striatum and a loss of synaptic markets^21^. In these conditions, release of glutamate is more likely to activate extrasynaptic N-methyl-D-aspartate receptors NMDARs), which are known to mediate apoptotic cascades^14^. Indeed, an increase in pro-apoptotic extrasynaptic NMDAR function occurs in YAC128 MSNs at the presymptomatic and symptomatic stages^6^. However, differences in extrasynaptic NMDARs from the direct and indirect pathways were not examined. This is important as studies have shown that MSNs of the indirect pathway are more vulnerable in HD^2^.

In the present study, we examined synaptic versus extrasynaptic electrophysiological changes induced by activation of AMPA and NMDA receptors in direct and indirect pathway MSNs of 16 month-old YAC128 mice and their age-matched wild type (WT) controls. Results revealed differential changes in synaptic and extrasynaptic activity of MSNs which could have important implications to understand the differential vulnerability of MSNs in these two striatal output pathways in HD.

## Materials and Methods

All experimental procedures were performed in accordance with the United States Public Health Service Guide for Care and Use of Laboratory Animals and were approved by the Institutional Animal Care and Use Committee at the University of California, Los Angeles (UCLA). Experiments were conducted in WT and YAC128 mice crossbred to FVB/N mice expressing enhanced green fluorescent protein (EGFP) under the control of the D1 or D2 dopamine receptor promoter. All mice were obtained from our breeding colonies at UCLA. Each experimental or control group consisted of 5-10 mice.

Detailed procedures for mouse slice preparation and electrophysiology have been published previously^4^. Briefly, the mice were deeply anaesthetized with isoflurane and decapitated. The brains were quickly removed and placed in oxygenated ice-cold low-Ca^2+ ^artificial cerebrospinal fluid (ACSF) containing (in mM): 130 NaCl, 3 KCl, 1.25 NaH_2_PO_4_, 26 NaHCO_3_, 5 MgCl_2_, 1 CaCl_2_, and 10 glucose. Coronal slices (350 μm) were cut and transferred to an incubating chamber containing ACSF (with 2 mM CaCl_2_ and 2 mM MgCl_2_) oxygenated with 95% O_2_-5% CO_2_ (pH 7.2-7.4, 290-310 mOsm, 25±2°C).

After 1 h incubation, direct and indirect pathway MSNs were selected for recordings. Cells were visualized using a 40x water-immersion lens on an Olympus microscope (BX50WI) equipped with differential interference contrast optics and fluorescence. After identifying MSNs with infrared videomicroscopy (QICAM-IR Fast 1394, Qimaging), the filter was switched to fluorescence to determine whether the cell was labeled with EGFP. The digitized infrared image was superimposed over the fluorescence image, and electrophysiological recordings proceeded only if the cell identified with infrared light overlapped with EGFP fluorescence.

All experiments were performed in voltage clamp mode, with patch electrodes (3-5 MΩ) filled with the following internal solution (in mM): 130 Cs-methanesulfonate, 10 CsCl, 4 NaCl, 1 MgCl_2_, 5 MgATP, 5 EGTA, 10 HEPES, 0.5 GTP, 10 phosphocreatine, (pH 7.25–7.3, osmolality, 280–290 mOsm). QX-314 (4 mM) was added to the intracellular solution to prevent Na^+ ^channel activation when stimulating or holding the membrane at depolarized potentials.

Synaptic stimulation: To assess input-output functions, a monopolar glass electrode (impedance 1 MΩ) was placed in the corpus callosum 200-300 μm from the MSN. To examine AMPAR-mediated currents, stimuli of increasing intensities (0.01-0.2 mA) were applied every 10 s, while for NMDAR-mediated currents stimuli were applied every 20 s. To record AMPAR-mediated currents, the membrane potential was clamped at -70mV in the presence of bicuculline (BIC, 10 μM). NMDAR-mediated currents were recorded at a holding potential of +40 mV in the presence of BIC (10 μM) and 6-Cyano-7nitroquinoxaline-2,3-dione (CNQX, 10 μM), which blocks AMPAR-mediated currents. Glycine (20 μM) and strychnine (10 μM) also were included to augment NMDAR function and block inhibitory glycine receptors, respectively^6^. DL-threo-β-Benzyloxyaspartic acid (DL-TBOA, 30 μM), a blocker of excitatory amino acid transporters, also was used to promote spillover of glutamate from synaptic receptors onto extrasynaptic NMDARs^6^. At this concentration, the blocker has no direct effects on NMDARs^22^. For this experiment, the current intensity that evoked 75% of the peak amplitude response was used. Peak amplitude, area, and decay time were determined using ClampFit software. Decay times were assessed using the 90-10% portion of the decay phase, where 100% was the peak amplitude and 0% was at baseline. Thus, the decay time is the absolute time measurement of the decay phase of the response between 90% and 10% peak amplitude values.

Values in figures and text are means ± SEMs. Differences between group means were assessed with appropriate *t*-tests and two-way analyses of variance (ANOVA) with one repeated measure, followed by Bonferroni *post hoc* tests. Differences were considered statistically significant if *p*<0.05.

## Results

**Evoked excitatory currents were decreased in YAC128 direct and indirect pathway MSNs.**

We first examined evoked AMPAR- and NMDAR-mediated currents in WT and YAC128 direct and indirect pathway MSNs in response to increasing stimulus intensities (Fig. 1). In YAC128 direct pathway MSNs (n=9), there was a significant decrease in peak amplitude of evoked AMPAR-mediated currents compared to WTs (n=8) (Genotype: *F*(1,15)=5.029, *p*=0.04; Interaction of genotype and stimulation intensity: *F*(6,90)=2.952, *p*=0.011) (Fig. 1B, left). Similarly, YAC128 indirect pathway MSNs (n=10) displayed significant decreases in peak amplitude of evoked AMPAR-mediated currents compared to WTs (n=15) (Genotype: *F*(1,20)=7.877, *p*=0.011; Interaction of genotype and stimulation intensity: *F*(6,120)=4.200, *p*<0.001) (Fig. 1B, right). Post-hoc analysis revealed that decreases in AMPAR- mediated currents were significant at 0.06-0.20 mA stimulation intensities (*p*=0.05-0.008) for direct pathway MSNs and at the 0.04-0.20 mA intensities (*p*=0.019-0.001) for indirect pathway MSNs. Peak NMDAR-mediated currents also were significantly decreased for direct pathway MSNs from YAC128 mice (Genotype: *F*(1,17)=5.285, *p*=0.034; Interaction of genotype and stimulation intensity: *F*(6,102)=4.874, *p*<0.001) (Fig. 1D, left). *Post hoc* analysis revealed that decreases in NMDAR-mediated currents were significant at 0.06-0.20 mA stimulation intensities (*p*=0.017-0.004). There was a non-significant but strong trend for a decrease in peak NMDAR-mediated currents between WT and YAC128 indirect pathway MSNs (Genotype: *F*(1,23)=3.844, *p*=0.062; Interaction of genotype and stimulation intensity: *F*(6,138)=1.955, *p*<0.076). The* post hoc* analysis revealed a significant difference in the peak NMDAR-mediated currents at the 0.04 mA stimulation intensity (*t*=2.787, *p*=0.008) (Fig. 1D).

**AMPAR- and NMDAR-mediated currents were significantly reduced in direct and indirect pathway MSNs in YAC128 mice.**
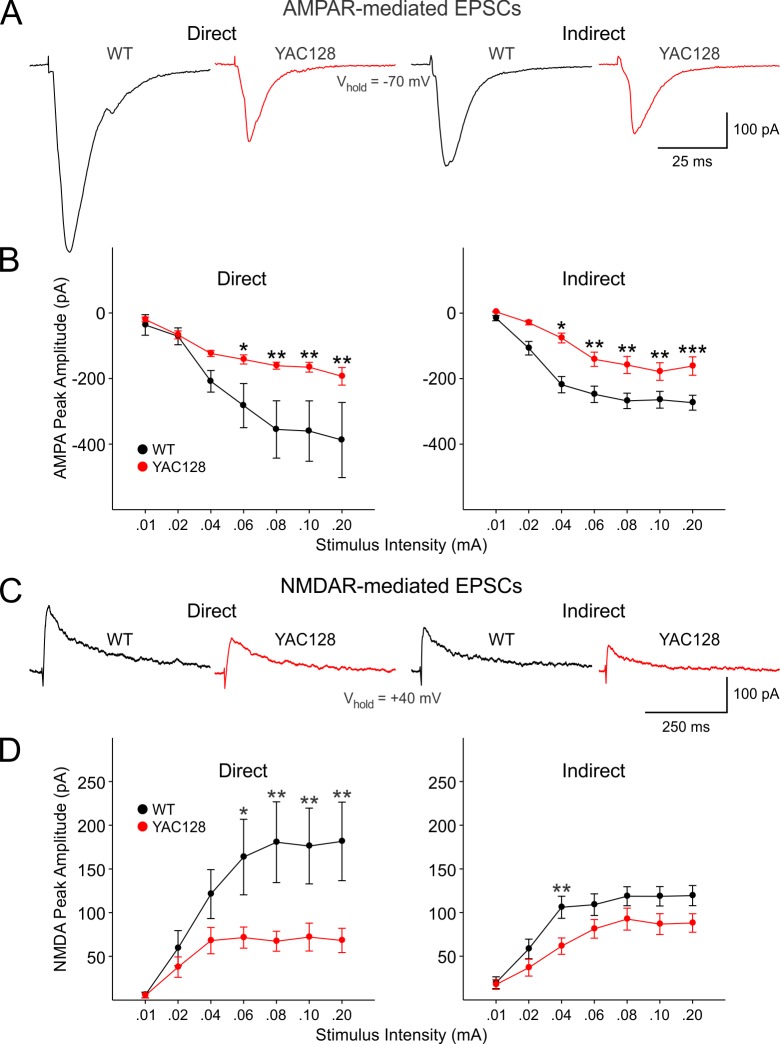
**A**: Typical traces of AMPAR- mediated currents in WT and YAC128 direct and indirect pathway MSNs evoked at the 0.10 mA stimulus intensity are shown. **B**: Input-output functions of evoked AMPAR-mediated currents were significantly decreased in both direct and indirect pathway MSNs of YAC128 mice. **C**: Typical traces of NMDAR-mediated currents in WT and YAC128 direct and indirect pathway MSNs evoked at 0.10 mA stimulus intensity are shown. **D**: Input-output functions of evoked NMDAR-mediated currents also were significantly reduced in both direct and indirect pathway MSNs from YAC128 mice. In this and other figures * indicates p<0.05, ** indicates p<0.01, *** indicates p<0.001. See text for details of statistics.

There were no significant differences in peak AMPAR- or NMDAR-mediated currents between direct or indirect pathway MSNs from either WT or YAC128 mice. Furthermore, there were no differences in NMDA/AMPA ratios among direct and indirect pathway MSNs in WT and YAC128 mice (0.51±0.074 vs. 0.44±0.08 for WTs and YAC128 from direct pathway MSNs, respectively, p=0.542; 0.45±0.047 vs. 0.56±0.056 for WTs and YAC128 from indirect pathway MSNs, respectively, p=0.135).

**Glutamate transport block revealed alterations in extrasynaptic NMDAR-mediated responses in YAC128 MSNs.**

We used DL-TBOA (30 μM), a glutamate transport blocker which promotes activation of extrasynaptic NMDARs through glutamate spillover, to assess differences in evoked NMDAR-mediated currents in direct and indirect pathway MSNs. We examined alterations in four indices, peak amplitude, decay time, response area and response area normalized by peak response separately for direct or indirect pathway MSNs (Fig.2). An increase in the contribution from extrasynaptic NMDA receptors would be shown by changes in peak amplitude, decay time and response area of the currents. We only tested the effects of TBOA at the stimulus intensity that produced 75% of the maximum response for each neuron. In WTs for both direct and indirect pathway MSNs, peak amplitude decreased significantly in TBOA (*t*=2.177, *p*=0.047; *t*=2.684, *p*=0.016 for direct and indirect pathway MSNs, respectively) (Fig. 2B). In both direct and indirect pathway MSNs, almost all neurons (6/7 and 9/10 for direct and indirect pathway MSNs, respectively) showed a decrease in the peak response amplitude although for each group there was considerable variation in the absolute amplitudes of the responses of the neurons. In contrast, in direct pathway MSNs of YAC128 mice, peak amplitude increased significantly (*t*=4.128, *p*=0.001; 8/9 neurons) while in indirect pathway MSNs peak amplitude decreased significantly (*t*=6.017, *p*<0.001; 7/7 neurons) (Fig. 2B).

Decay time increased significantly for both direct and indirect pathway MSNs in WTs and YAC128 mice (*t*=2.817, *p*=0.014;* t*=3.131, *p*=0.006 for direct (7/7 neurons) and indirect pathway (11/11 neurons) MSNs in WTs, respectively; *t*=4.601, *p*<0.001;* t*=5.153, *p*<0.001 for direct (9/9 neurons) and indirect pathway (7/7 neurons) MSNs in YAC128 mice, respectively) (Fig. 2C). Response area increased significantly in direct pathway MSNs in only YAC128 mice (*t*=3.143, *p*=0.007; 9/9 neurons) (Fig. 2D). In contrast, in indirect pathway MSNs response area increased significantly in both WTs and YAC128 mice (*t*=3.371, *p*<0.004 for WTs, 11/11 neurons; *t*=3.352, *p*=0.004 for YAC128, 7/7 neurons).

Because DL-TBOA affected direct and indirect pathway MSN peak values in opposing ways, the area was normalized by peak amplitude ((pA*ms)/pA) to verify a difference in the density of extrasynaptic NMDARs between direct and indirect pathway MSNs^6^. In this way the relative area could be compared without the confounding influence of the peak amplitude. Normalized response area increased significantly in all groups (*t*=5.090, *p*<0.001;* t*=2.64, *p*=0.017 for direct and indirect pathway MSNs in WTs, respectively; *t*=4.125, *p*=0.001; *t*=6.622, *p*<0.001 for direct and indirect pathway MSNs in YAC128, respectively) (Fig. 2E). In addition, the normalized area increase was significantly greater for indirect pathway MSNs in YAC128 mice compared to WTs (*t*=3.649, *p*=0.001). These findings indicate that both response amplitude and decay are affected by TBOA but in slightly different ways in direct and indirect pathway MSNs in YAC128 mice.

**DL-TBOA alters peak amplitude, decay, area and relative area of direct and indirect pathway MSNs of WT and YAC128 mice.**
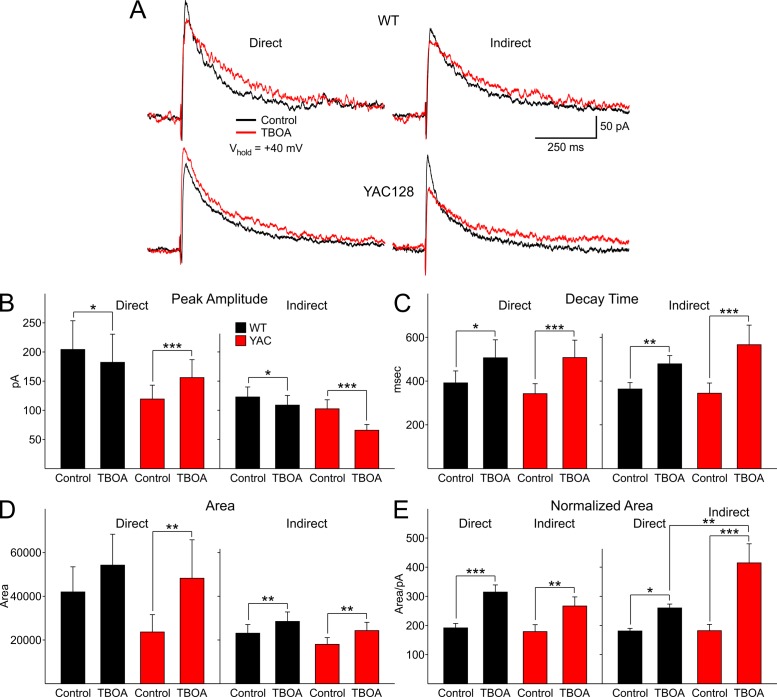
A: Typical traces of NMDAR-mediated currents in WT and YAC128 direct and indirect pathway MSNs evoked at 75% of the maximal stimulus intensity showing alterations in peak amplitudes and decay times. B: Peak amplitude is differentially altered in direct and indirect pathway MSNs by DL-TBOA. Peak amplitude increases in direct pathway MSNs but decreases in indirect pathway MSNs in YAC128 mice. C: In DL-TBOA response decay time increases in direct and indirect pathway MSNs in both WT and YAC128 mice. D: In DL-TBOA, response area increases only in direct pathway MSNs in YAC128 mice and in indirect pathway MSNs in both WT and YAC128 mice. E: In DL-TBOA, normalized area increases in all groups but displays the greatest increase in indirect pathway MSNs in YAC128 mice.

To more specifically compare the effects of TBOA in direct and indirect pathway MSNs, changes induced by TBOA were converted to percentages (Fig. 3). In direct pathway MSNs, TBOA decreased the evoked NMDAR peak current in WTs but increased it in YAC128 mice (*t*=6.449,* p*<0.001) (Fig. 3A). In indirect pathway MSNs TBOA decreased the peak in both WT and YAC128 mice, but the reduction was significantly larger in YAC128 D2 MSNs (*t*=3.323, *p*=0.002) (Fig. 3A). The difference in peak amplitude between direct and indirect pathway MSNs in YAC128 mice also was significant (*t*=9.150, *p*<0.001). Decay time was significantly elevated in indirect YAC128 MSNs compared to WTs (*t*=3.601, *p*=0.001) (Fig. 3B) and also was significantly greater in indirect pathway MSNs compared to direct pathway MSNs in YAC128 mice (*t*=2.258, *p*=0.031). Area was significantly larger in YAC128 direct pathway MSNs compared to area of WTs (*t*=3.482, *p*=0.002) (Fig. 3C). The increased area of indirect pathway MSNs approached statistical significance (*t*=1.829, *p*=0.077). The increased area of direct versus indirect pathway MSNs was significantly greater in only the YAC128 mice (*t*=2.353, *p*<0.025). There was a significant increase in relative area in indirect pathway MSNs in YAC128 mice compared to WTs (*t*=5.012, *p*<0.001) as well as a significant increase in indirect pathway MSNs compared to direct pathway MSNs but only in MSNs in YAC128 mice (*t*=4.158, *p*<0.001) (Fig. 3D).

**Differential effects of DL-TBOA on direct and indirect pathway MSNs exemplified by percentage changes.**
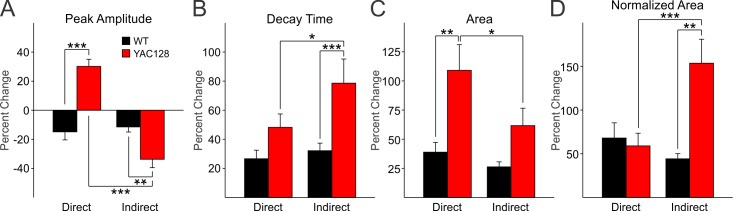
**A**: Percentage changes in peak response amplitude were differentially altered in direct and indirect pathway MSNs of YAC128 mice. **B**: Percentage changes in decay times were greater in indirect pathway compared to direct pathway MSNs from YAC128 mice. **C**: Percentage change in response area were greatest in direct pathway MSNs of YAC128 mice. **D**: Normalized area increases were greatest in indirect pathway MSNs of YAC128 mice.

## Discussion

The present study demonstrated decreases in evoked excitatory postsynaptic currents mediated by AMPARs and NMDARs in YAC128 direct and indirect pathway MSNs. Furthermore, in contrast to the downregulation of synaptic activity, NMDAR-mediated responses evoked in the presence of TBOA were increased in both direct and indirect pathway MSNs of YAC128 mice, but in slightly different ways. In direct pathway MSNs, response increases were mainly due to higher amplitudes while in indirect pathway MSNs, the increase was due to longer decay times. This outcome indicates differential changes in synaptic versus extrasynaptic NMDA receptors in both direct and indirect pathway MSNs from these older, symptomatic YAC128 mice.

We previously reported a progressive disconnection of striatal MSNs from their glutamatergic inputs in various models of HD^7^^,^ 8. In 12 month-old YAC128 mice we also showed that the reduction in the spontaneous excitatory postsynaptic current frequency was greater in direct than indirect pathway MSNs but we did not examine AMPAR and NMDAR responses separately and our results were based on AMPAR-mediated responses^4^. The reduction in evoked synaptic responses agrees with previous studies showing decreases in glutamate release as well as AMPAR- and NMDAR-mediated currents in symptomatic YAC128 mice^9^. The reduction in the amplitude of evoked responses indicates that there is a loss of glutamatergic inputs onto MSNs in YAC128 mice at this age for MSNs originating both pathways in HD. The loss of glutamatergic inputs may be due to pre- and postsynaptic mechanisms. In YAC128 mice a reduction in spine density has been observed^10^. While the reduction in spine density could be caused by pre- and post-synaptic mechanisms, recent studies demonstrate a critical role of postsynaptic NMDA receptors containing GluN3A subunits in HD spine loss^11^.

In contrast to the reduction in evoked synaptic currents, NMDA responses evoked in the presence of TBOA were increased. Previous studies demonstrated an increase in the extrasynaptic NMDAR-mediated responses in young YAC128 mice^6^. However, differences between direct and indirect pathway MSNs were not examined. The present study not only confirms that this increase in extrasynaptic NMDAR-mediated responses occurs in old YAC128 mice, but also demonstrates that it occurs in both direct and indirect pathway MSNs though it appears to be mechanistically due to differing underlying effetcs. Direct pathway MSNs show a larger increase in peak amplitude while indirect pathway MSNs display a larger increase in normalized area due to a longer decay time. This latter effect could suggest that MSNs of the indirect pathway are more sensitive to changes in glutamate transport, thus contributing to their increased vulnerability in HD.

NMDARs are composed by GluN2A and GluN2B subunits. GluN2A subunits are thought to be restricted mainly to the synaptic site^12^, while the GluN2B subunits are located on synaptic and extrasynaptic sites^13^. It has been suggested that the activation of the synaptic NMDARs is neuroprotective, while activation of extrasynaptic NMDA receptors stimulates a pro-apoptotic cellular pathway^14^. Alterations in NMDAR subunit trafficking is thought to contribute to increased density of GluN2B subunits at extrasynaptic sites in HD^15^^,^ 16. In a recent study employing co-culture of cortical and striatal MSNs from YAC128 mice, a larger extrasynaptic NMDAR-mediated current occurred in association with an increase in GluN2B subunit expression at the cellular surface compared with the co-culture obtained from WT mice^6^^,^ ^17^. In addition, low-dose memantine, a blocker of GluN2B-containing NMDARs at extrasynaptic sites, ameliorates neuropathological and behavioral manifestations of HD in YAC128 mice^6^^,^ 18. In co-cultured YAC128 MSNs, low doses of memantine and ifenprodil attenuated extrasynaptic NMDAR-mediated neuronal apoptosis^17^.

In conclusion, the present results showed that the glutamatergic inputs to MSNs are decreased in 16-month old YAC128 mice. This study also demonstrates that in late stage HD, NMDA responses evoked in both direct and indirect pathway MSNs in the presence of TBOA are significantly increased in YAC128 mice and the parameters for the increase are different. Direct pathway MSNs in YAC128 mice display increases in peak amplitude while indirect pathway MSNs in YAC128 mice display increases in normalized area due to significantly slower decay times. These results suggest that alterations in glutamate reuptake have different effects on indirect compared with direct pathway MSNs and highlight previous studies showing that MSNs of both pathways are differentially affected in mouse models of HD^19^^,^ 20.

## References

[1] G. Bates, P.S. Harper, L. Jones (Eds.), Huntington's disease, Oxford University Press, Oxford ; New York, 2002, xvi, 558 p.

[2] Slow EJ, van Raamsdonk J, Rogers D, Coleman SH, Graham RK, Deng Y, Oh R, Bissada N, Hossain SM, Yang YZ, Li XJ, Simpson EM, Gutekunst CA, Leavitt BR, Hayden MR. Selective striatal neuronal loss in a YAC128 mouse model of Huntington disease. Hum Mol Genet. 2003 Jul 1;12(13):1555-67. PubMed PMID:12812983. 12812983 10.1093/hmg/ddg169

[3] Ferrante RJ. Mouse models of Huntington's disease and methodological considerations for therapeutic trials. Biochim Biophys Acta. 2009 Jun;1792(6):506-20. PubMed PMID:19362590. 19362590 10.1016/j.bbadis.2009.04.001 PMC2693467

[4] André VM, Cepeda C, Fisher YE, Huynh M, Bardakjian N, Singh S, Yang XW, Levine MS. Differential electrophysiological changes in striatal output neurons in Huntington's disease. J Neurosci. 2011 Jan 26;31(4):1170-82. PubMed PMID:21273402. 21273402 10.1523/JNEUROSCI.3539-10.2011 PMC3071260

[5] André VM, Fisher YE, Levine MS. Altered Balance of Activity in the Striatal Direct and Indirect Pathways in Mouse Models of Huntington's Disease. Front Syst Neurosci. 2011;5:46. PubMed PMID:21720523. 21720523 10.3389/fnsys.2011.00046 PMC3118454

[6] Milnerwood AJ, Gladding CM, Pouladi MA, Kaufman AM, Hines RM, Boyd JD, Ko RW, Vasuta OC, Graham RK, Hayden MR, Murphy TH, Raymond LA. Early increase in extrasynaptic NMDA receptor signaling and expression contributes to phenotype onset in Huntington's disease mice. Neuron. 2010 Jan 28;65(2):178-90. PubMed PMID:20152125. 20152125 10.1016/j.neuron.2010.01.008

[7] Cepeda C, Hurst RS, Calvert CR, Hernández-Echeagaray E, Nguyen OK, Jocoy E, Christian LJ, Ariano MA, Levine MS. Transient and progressive electrophysiological alterations in the corticostriatal pathway in a mouse model of Huntington's disease. J Neurosci. 2003 Feb 1;23(3):961-9. PubMed PMID:12574425. 12574425 10.1523/JNEUROSCI.23-03-00961.2003 PMC6741903

[8] Cummings DM, Cepeda C, Levine MS. Alterations in striatal synaptic transmission are consistent across genetic mouse models of Huntington's disease. ASN Neuro. 2010 Jun 18;2(3):e00036. PubMed PMID:20585470. 20585470 10.1042/AN20100007 PMC2888168

[9] Joshi PR, Wu NP, André VM, Cummings DM, Cepeda C, Joyce JA, Carroll JB, Leavitt BR, Hayden MR, Levine MS, Bamford NS. Age-dependent alterations of corticostriatal activity in the YAC128 mouse model of Huntington disease. J Neurosci. 2009 Feb 25;29(8):2414-27. PubMed PMID:19244517. 19244517 10.1523/JNEUROSCI.5687-08.2009 PMC2670193

[10] Graham RK, Pouladi MA, Joshi P, Lu G, Deng Y, Wu NP, Figueroa BE, Metzler M, André VM, Slow EJ, Raymond L, Friedlander R, Levine MS, Leavitt BR, Hayden MR. Differential susceptibility to excitotoxic stress in YAC128 mouse models of Huntington disease between initiation and progression of disease. J Neurosci. 2009 Feb 18;29(7):2193-204. PubMed PMID:19228972. 19228972 10.1523/JNEUROSCI.5473-08.2009 PMC2729178

[11] Marco S, Giralt A, Petrovic MM, Pouladi MA, Martínez-Turrillas R, Martínez-Hernández J, Kaltenbach LS, Torres-Peraza J, Graham RK, Watanabe M, Luján R, Nakanishi N, Lipton SA, Lo DC, Hayden MR, Alberch J, Wesseling JF, Pérez-Otaño I. Suppressing aberrant GluN3A expression rescues synaptic and behavioral impairments in Huntington's disease models. Nat Med. 2013 Aug;19(8):1030-8. PubMed PMID:23852340. 23852340 10.1038/nm.3246 PMC3936794

[12] Thomas CG, Miller AJ, Westbrook GL. Synaptic and extrasynaptic NMDA receptor NR2 subunits in cultured hippocampal neurons. J Neurophysiol. 2006 Mar;95(3):1727-34. PubMed PMID:16319212. 16319212 10.1152/jn.00771.2005

[13] Cull-Candy S, Brickley S, Farrant M. NMDA receptor subunits: diversity, development and disease. Curr Opin Neurobiol. 2001 Jun;11(3):327-35. PubMed PMID:11399431. 11399431 10.1016/s0959-4388(00)00215-4

[14] Hardingham GE, Bading H. Synaptic versus extrasynaptic NMDA receptor signalling: implications for neurodegenerative disorders. Nat Rev Neurosci. 2010 Oct;11(10):682-96. PubMed PMID:20842175. 20842175 10.1038/nrn2911 PMC2948541

[15] Fan MM, Raymond LA. N-methyl-D-aspartate (NMDA) receptor function and excitotoxicity in Huntington's disease. Prog Neurobiol. 2007 Apr;81(5-6):272-93. PubMed PMID:17188796. 17188796 10.1016/j.pneurobio.2006.11.003

[16] Levine MS, Cepeda C, André VM. Location, location, location: contrasting roles of synaptic and extrasynaptic NMDA receptors in Huntington's disease. Neuron. 2010 Jan 28;65(2):145-7. PubMed PMID:20152121. 20152121 10.1016/j.neuron.2010.01.010 PMC3119569

[17] Milnerwood AJ, Kaufman AM, Sepers MD, Gladding CM, Zhang L, Wang L, Fan J, Coquinco A, Qiao JY, Lee H, Wang YT, Cynader M, Raymond LA. Mitigation of augmented extrasynaptic NMDAR signaling and apoptosis in cortico-striatal co-cultures from Huntington's disease mice. Neurobiol Dis. 2012 Oct;48(1):40-51. PubMed PMID:22668780. 22668780 10.1016/j.nbd.2012.05.013

[18] Okamoto S, Pouladi MA, Talantova M, Yao D, Xia P, Ehrnhoefer DE, Zaidi R, Clemente A, Kaul M, Graham RK, Zhang D, Vincent Chen HS, Tong G, Hayden MR, Lipton SA. Balance between synaptic versus extrasynaptic NMDA receptor activity influences inclusions and neurotoxicity of mutant huntingtin. Nat Med. 2009 Dec;15(12):1407-13. PubMed PMID:19915593. 19915593 10.1038/nm.2056 PMC2789858

[19] Albin RL, Reiner A, Anderson KD, Dure LS 4th, Handelin B, Balfour R, Whetsell WO Jr, Penney JB, Young AB. Preferential loss of striato-external pallidal projection neurons in presymptomatic Huntington's disease. Ann Neurol. 1992 Apr;31(4):425-30. PubMed PMID:1375014. 1375014 10.1002/ana.410310412

[20] Reiner A, Albin RL, Anderson KD, D'Amato CJ, Penney JB, Young AB. Differential loss of striatal projection neurons in Huntington disease. Proc Natl Acad Sci U S A. 1988 Aug;85(15):5733-7. PubMed PMID:2456581. 2456581 10.1073/pnas.85.15.5733 PMC281835

[21] Cepeda C, Wu N, André VM, Cummings DM, Levine MS. The corticostriatal pathway in Huntington's disease. Prog Neurobiol. 2007 Apr;81(5-6):253-71. PubMed PMID:17169479. 17169479 10.1016/j.pneurobio.2006.11.001 PMC1913635

[22] Jabaudon D, Shimamoto K, Yasuda-Kamatani Y, Scanziani M, Gähwiler BH, Gerber U. Inhibition of uptake unmasks rapid extracellular turnover of glutamate of nonvesicular origin. Proc Natl Acad Sci U S A. 1999 Jul 20;96(15):8733-8. PubMed PMID:10411944. 10411944 10.1073/pnas.96.15.8733 PMC17585

