# Correction: Differential Synaptic and Extrasynaptic Glutamate-Receptor Alterations in Striatal Medium-Sized Spiny Neurons of Aged YAC128 Huntington's Disease Mice

**DOI:** 10.1371/currents.hd.2f36af340a70a0d248b48d4cc9c3e58e

**Published:** 2017-07-11

**Authors:** PLOS Currents

## Correction

There is an error in the second author's name. The correct name is Elizabeth A. Wang. The correct citation is Botelho EP, Wang EA, Chen JY, Holley S, Andre V, Cepeda C, Levine MS. Differential Synaptic and Extrasynaptic Glutamate-Receptor Alterations in Striatal Medium-Sized Spiny Neurons of Aged YAC128 Huntington’s Disease Mice. PLOS Currents Huntington Disease. 2014 May 21 . Edition 1. doi: 10.1371/currents.hd.34957c4f8bd7cb1f5ec47381dfc811c3.

## Reference

Botelho EP, Wang E, Chen JY, Holley S, Andre V, Cepeda C, Levine MS. Differential Synaptic and Extrasynaptic Glutamate-Receptor Alterations in Striatal Medium-Sized Spiny Neurons of Aged YAC128 Huntington’s Disease Mice. PLOS Currents Huntington Disease. 2014 May 21 . Edition 1. doi: 10.1371/currents.hd.34957c4f8bd7cb1f5ec47381dfc811c3.

